# Further Results on the Proportional Vitalities Model

**DOI:** 10.3390/e23091201

**Published:** 2021-09-11

**Authors:** Mohamed Kayid

**Affiliations:** Department of Statistics and Operations Research, College of Science, King Saud University, P.O. Box 2455, Riyadh 11451, Saudi Arabia; drkayid@ksu.edu.sa

**Keywords:** vitality function, hazard rate order, mean residual live order, closure preservation

## Abstract

In contrast to many survival models such as proportional hazard rates and proportional mean residual lives, the proportional vitalities model has also been introduced in the literature. In this paper, further stochastic ordering properties of a dynamic version of the model with a random vitality growth parameter are investigated. Examples are presented to illustrate different established properties of the model. Potentials for inference about the parameters in proportional vitalities model with possibly time-varying effects are also argued and discussed.

## 1. Introduction

In survival analysis modelling time-to-failure data, numerous strategies have been identified over the years. The Cox proportional hazards (CPH) model proposed by Cox [1] has played a central role in lifetime data analysis in different applied situations. Due to its unique and fascinating properties, the CPH model stands head-and-shoulders above all other survival models. In the CPH model, the unique effect of a subject increase in a covariate is multiplicative with respect to the hazard rate. The proportional mean residual life (PMRL) model has been proposed by Oakes and Dasu [2] to model lifetime events. While the former model is more applicable and attractive in the sense of doing computations and calculating likelihood functions based on the available sample data, the latter model gives a survival function that is appeared to be more complicated. For this reason, in the PMRL model, it is not easy to implement inferential procedures to estimate the parameters of the model, specifically because the likelihood function of the model does not have a simple expression. In such cases, comparisons of the models using some quantitative methods are possible. These include stochastic orderings and aging behaviors of lifetime distributions, which can be used to discover some unique features of a model, which in turn further illustrate the nature and the properties of a model. Shanthikumar [3] introduced the proportional equilibrium rates model in a discrete setting. In recent decades, some researchers investigated the performance of the CPH model and the PMRL model in terms of a few closure traits and preservation properties with respect to some stochastic orderings and aging properties (see, e.g., Gupta and Kirmani [4], Nanda et al. [5], Kochar and Xu [6], Nanda et al. [7], and Nanda and Das [8]).

For a non-negative random variable (rv) *X* with absolutely continuous cumulative distribution function (cdf) *F* and probability density function (pdf) *f* with survival function $\bar{F}\equiv 1-F$, the hazard rate (hr) function of *X* is defined as

$$h_{X}\left(t\right)=\lim_{\delta \to 0^{+}}\frac{1}{\delta }P\left(X<t+\delta |X>t\right)=\frac{f(t)}{\bar{F}\left(t\right)},$$

for all *t* for which the denominator is positive. The hr function measures relative probabilities of sudden deaths. For more general distributions (i.e., not necessarily absolutely continuous), the mean residual life (mrl) function of *X* is expressed as

$$M_{X}\left(t\right)=E\left(X-t|X\geq t\right)=\frac{1}{\bar{F}\left(t\right)}\int_{t}^{\infty }\bar{F}\left(x\right)\;dx,$$

which is well-defined for all t≥0 with the additional requirement that F(t)<1. To provide a fresh perspective on $M_{X}$ and its properties, the vitality function of *X* has been defined as an intrinsically non-decreasing function, by

$$V_{X}\left(t\right)=E\left(X|X\geq t\right)=\frac{\int_{t}^{\infty }x\;dF\left(x\right)}{\bar{F}\left(t\right)},\;\;\mathrm{for}\;\mathrm{all}\;t:F\left(t\right)<1.$$

It is obvious that $V_{X}$ is closely related to the mrl of X, as

(1) $$V_{X}\left(t\right)=M_{X}\left(t\right)+t.$$

The vitality function, which was considered by Kupka [9] as a derivation of a more general measure, characterizes the underlying cdf uniquely as the mrl function does. Several authors used this quantity to establish some characterizations of the underlying cdf (see, e.g., Gupta [10], Ruiz and Navarro [11], Gupta and Kirmani [12], Midhu et al. [13], and the references therein). Estimation of the vitality function and the corresponding inferential procedures have also captured the attention of some researches (cf. Ruiz and Guillamón [14] and Guillamón et al. [15]). Oakes and Dasu [2] proposed first the PMRL model as

(2) $$M\left(t|\theta \right)=\theta M_{0}\left(t\right),\;t\geq 0,$$

where θ is a positive parameter, M(·|θ) is the mrl function of a response variable, and $M_{0}$ is the baseline mrl function. Then, Zahedi [16] highlighted the role of this model to be played by a regression model. This way, the effect of data in changing the behavior of a baseline mrl function appears in terms of some regression coefficients. Estimation procedures for coefficients of the regression PMRL model have been conducted by Maguluri and Zhang [17] and Chen et al. [18]. Recently, Shrahili et al. [19] proposed the proportional vitalities (PVIT) model as an alternative to the PMRL model and also the CPH model. Unlike the prevalent CPH and PMRL models, in the PVIT model, the sf and the pdf of the dependent random variable do not have an explicit closed form.

According to Shrahili et al. [19] the PVIT model is identified by

(3) $$v\left(t|\xi \right)=\xi v_{0}\left(t\right),\;t\geq 0,$$

where ξ>0 is the vitality growth parameter with a time-dependent domain so that $\xi \geq t/v_{0}\left(t\right)$, for all t≥0, the function v(·|ξ) is the vitality function of the population or the dependent variable and $v_{0}$ is the baseline vitality function. The model (3) is a partial model but it stands valid and qualified under more general circumstances.

In this paper, the model (3) is considered to investigate its further stochastic properties using the theory of stochastic orders. Random effects of the vitality growth is specially under consideration, since it provides a development to the model. Time-dependent constants of proportionality in the model (3) is also considered to extend the model. The scenario to present the contribution is as follows. In Section 2, the state of art and some recent literature in the context of PMR and additive mean residual life (AMRL) model is given. In Section 3, the unconditional sf and the unconditional pdf are obtained in order to present a method a method for measuring probabilities in the PVIT model. Stochastic orderings of random variables in the model are also studied in that section. In Section 4, the model (3) is developed for the case where ξ is replaced with a function allowed to be time-varying. In Section 5, inference about the parameters of the time-dependent PVIT model in a special case is made. Finally, in Section 6, the paper is summarized and concluded with further remarks and expectations for a future study.

## 2. State of Art and Recent Literature Review

To present the state of scientific development in the context of recent survival models, we consider the works accomplished in the context of the PMRL model and the AMRL model that are closely related to the PVIT model. In fact, the PVIT model is a special case of an additive-multiplicative mrl model as stated in the proof of Theorem 2 in Shrahili et al. [19]. Recently, Nair et al. [20] carried out a reliability study of the PMRL model in the frame of quantile functions. Statistical inference in the PMRL model has recently been followed by Lee et al. [21] and Wang and Chen [22], among others, while some inferential studies in the AMRL model have been tackled by He et al. [23] and Wu et al. [24].

## 3. The Unobserved Vitality Growth ξ

Frailty models have gathered the attention of many researchers in recent decades (see, e.g., Cha and Finkelstein [25] and Misra and Francis [26]). In this section, to consider the possibility to equip the PVIT model with random variations of the growth parameter, we develop the random effect proportional vitalities model, which extends the model given earlier in (3) to the case where ξ is a realization of or an observation on a random variable.

Denote by *X* the random variable that has the vitality function $v_{0}$ in (3). The function $v_{0}\left(t\right)=E\left(X|X>t\right)$ is then called the baseline vitality function. Denote by Ξ the random vitality growth, which is assumed to have cdf *G* and pdf *g*. Clearly, the random variable Ξ is non-negative. It has been taken for granted that there is a realization of Ξ that equals ξ. Given that Ξ=ξ, the conditional vitality function of the population is obtained as $v\left(t|\xi \right)=\xi v_{0}\left(t\right)$ for all t≥0. Shrahili et al. [19] obtained the conditional cdf with corresponding conditional vitality function $v\left(t|\xi \right)=\xi v_{0}\left(t\right)$ as

(4) $$\bar{F}\left(t\;|\;\xi \right)=\exp\left(-\int_{0}^{t}\frac{\xi v_{0}^{\prime }\left(x\right)}{\xi v_{0}\left(x\right)-x}dx\right),\;t\geq 0,$$

and

(5) $$f\left(t\;|\;\xi \right)=\frac{\xi v_{0}^{\prime }\left(t\right)}{\xi v_{0}\left(t\right)-t}\exp\left(-\int_{0}^{t}\frac{\xi v_{0}^{\prime }\left(x\right)}{\xi v_{0}\left(x\right)-x}dx\right),\;t\geq 0.$$

To integrate the effect of random variable Ξ, we denote by *U* the unconditional random variable, which has sf ${\bar{F}}_{U}\left(t\right)=\int_{0}^{\infty }\bar{F}\left(t\;|\;\xi \right)g\left(\xi \right)d\xi$. By replacing $\bar{F}\left(t\;|\;\xi \right)$ from (4),

(6) $${\bar{F}}_{U}\left(t\right)=\int_{0}^{\infty }\exp\left(-\int_{0}^{t}\frac{\xi v_{0}^{\prime }\left(x\right)}{\xi v_{0}\left(x\right)-x}dx\right)g\left(\xi \right)\;d\xi ,\;t\geq 0.$$

The pdf of *U* is also $f_{U}\left(t\right)=\int_{0}^{\infty }f\left(t|\xi \right)g\left(\xi \right)d\xi$, which is obtained by substituting f(t|ξ) from (5) with

(7) $$f_{U}\left(t\right)=\int_{0}^{\infty }\frac{\xi v_{0}^{\prime }\left(t\right)}{\xi v_{0}\left(t\right)-t}\exp\left(-\int_{0}^{t}\frac{\xi v_{0}^{\prime }\left(x\right)}{\xi v_{0}\left(x\right)-x}dx\right)g\left(\xi \right)\;d\xi ,\;t\geq 0.$$

The connection of three random variables X,Ξ, and *U* is as follows. The random variable Ξ is the mixing factor upon which, when observed, the random variable *U* is connected with the random variable *X* through their vitality functions. The variation in Ξ is not independent of the variation in *X*, as will be illustrated in Remark 1. The variation in *X* is considered to be independent, but the variation in *U* depends on the amount of Ξ and further depends on the variation in *X*. In other words, when Ξ=ξ, then $v_{U|\Xi =\xi }\left(t\right)=E\left(U|U>t,\Xi =\xi \right)=\xi E\left(X|X>t\right).$ However, when Ξ is unspecified, the relationship between the vitality function of *U* and the vitality function of *X* is realized in Theorem 2. The model (3) when ξ is specified is called the individual level PVIT model.

**Remark** **1.** *In the individual level PVIT model in order to have a valid model, the amount of ξ will restrict the possibility of taking an arbitrary baseline cdf F in the model. The identity (3) is valid when $\xi >(t/v_{0}\left(t\right)),$ for all t≥0. For example, if ξ=1/2 and if F is a cdf with vitality function $v_{0}$ for which $(t/v_{0}\left(t\right))>1/2$ for some values t in the support of F, then, for these choices of ξ and F, the PVIT model in (3) is not valid. However, when ξ≥1, the assignment of F is independent of the value of ξ since $(t/v_{0}\left(t\right))\leq 1\leq \xi$ for all t≥0. In the context of the frailty model, this issue is more controversial because the density g in (6) may be considered dependent on time constraints. To describe a situation where this issue is eliminated, consider the case when there exists an c>0 for which $\xi \geq c\geq (t/v_{0}\left(t\right)),$ for all t≥0 in which ξ is set to be any possible outcome of *Ξ*. The pdf g of *Ξ* may, therefore, be independent of time since the support of *Ξ* does not depend on time in this case.*

Consider the random couple (U,Ξ) with joint pdf f(u,ξ) and joint sf $\bar{F}\left(u,\xi \right)$. The origin of the vitality v(t|ξ) may be

$$\begin{array}{cc} v(t|\xi ) & =E(U|U>t,\Xi =\xi )\\ \; & =\int_{t}^{\infty }\frac{uf(u,\xi )}{-\partial \bar{F}\left(t,\xi \right)/\partial \xi }du\\ \; & =\int_{t}^{\infty }\frac{uf(u|\xi )}{\bar{F}\left(t|\xi \right)}du\\ \; & =\int_{t}^{\infty }\frac{\bar{F}\left(u|\xi \right)}{\bar{F}\left(t|\xi \right)}du+t, \end{array}$$

where f(u|ξ) and F(u|ξ) are the conditional pdf and the conditional cdf of *U* given Ξ=ξ, respectively. In the sequel, T≡(U|Ξ=ξ) is taken as a random variable with pdf f(·|ξ) for a given ξ>0. The above equations authenticate that $v\left(t|\xi \right)=v_{T}\left(t\right)=m_{T}\left(t\right)+t$ where $v_{T}$ and $m_{T}$ are the vitality function and the mrl function of *T*, respectively. Therefore, as recognized in (6), *U* follows a mixture model with mixing cdf *G*.

The probability given in (6) is concerned with a random experiment in which the growth parameter Ξ is random. For the individual randomly chosen from the population, Ξ=ξ is recognized. The time-to-failure of the individual at that level of vitality growth then follows the sf (4). In statistical analysis of survival data, the sf in (6) and the pdf in (7) represent a mixture population consisting of individuals bearing a dynamic vitality growth.

The value of $\bar{F}(t$|ξ)=P(U>t|Ξ=ξ) is the probability to survive after the age *t* given that the randomly selected individual has a specified vitality growth ξ. In Bayesian statistics, the inference is carried out by the likelihood of a particular value of the unknown parameter given the known data, which is assumed to follow the mixture model. Therefore, we obtain the density function of the unknown growth parameter ξ given a single observation on *U*. To be more specific, given that U=t, the density of Ξ is refreshed as

(8) $$\begin{array}{cc} g(\xi \;|\;t) & =\frac{f(t\;|\;\xi )g(\xi )}{\int_{0}^{\infty }f\left(t\;|\;\xi \right)g\left(\xi \right)\;d\xi }\\ \; & =\frac{\left(\xi g\left(\xi \right)/\left(\xi v_{0}\left(t\right)-t\right)\right)\exp\left(-\int_{0}^{t}\frac{\xi v_{0}^{\prime }\left(x\right)}{\xi v_{0}\left(x\right)-x}dx\right)}{\int_{0}^{\infty }\left(\xi g\left(\xi \right)/\left(\xi v_{0}\left(t\right)-t\right)\right)\exp\left(-\int_{0}^{t}\frac{\xi v_{0}^{\prime }\left(x\right)}{\xi v_{0}\left(x\right)-x}dx\right)\;d\xi },\;t\geq 0. \end{array}$$

The density function of Ξ among survivors of age *t* is

(9) $$\begin{array}{cc} g(\xi \;|\;U>t) & =\frac{\bar{F}\left(t\;|\;\xi \right)g\left(\xi \right)}{\int_{0}^{\infty }\bar{F}\left(t\;|\;\xi \right)g\left(\xi \right)\;d\xi }\\ \; & =\frac{g\left(\xi \right)\exp\left(-\int_{0}^{t}\frac{\xi v_{0}^{\prime }\left(x\right)}{\xi v_{0}\left(x\right)-x}dx\right)}{\int_{0}^{\infty }g\left(\xi \right)\exp\left(-\int_{0}^{t}\frac{\xi v_{0}^{\prime }\left(x\right)}{\xi v_{0}\left(x\right)-x}dx\right)\;d\xi },\;t\geq 0. \end{array}$$

The density function of Ξ|U=t and the density function of Ξ|U>t have generally complicated forms, especially when the baseline vitality function $v_{0}$ is unknown so that solving the likelihood equation to derive the maximum likelihood estimates of ξ is rarely possible. That being so, it is better for investigating some stochastic ordering properties in terms of the posterior distribution of Ξ among individuals with a certain age point or/and survivors of a fixed age. We refer readers to Shaked and Shanthikumar [27] for definition of the likelihood ratio order ($\leq_{lr}$), the hazard rate order (≤hr) and the usual stochastic order ($\leq_{st}$) and their further properties. The bivariate non-negative function ψ is said to be totally positive of order 2 (denoted by $TP_{2}$) in the set *S* whenever for all $x_{1}\leq x_{2}$ and for all $y_{1}\leq y_{2}$ such that $(x_{i},y_{i})\in S,i=1,2$, it holds that $\psi \left(x_{1},y_{1}\right)\psi \left(x_{2},y_{2}\right)\geq \psi \left(x_{1},y_{2}\right)\psi \left(x_{2},y_{1}\right)$ (see, e.g., Karlin [28]).

**Theorem** **1.** *Let U have survival function (6). Then,*

*(i)*  *For any t≥0, (Ξ|$U=t)\leq_{lr}\;(\Xi$|U>t).*
*(ii)*  *For all $t_{2}\geq t_{1}\geq 0,$ (Ξ|$U>t_{1})\leq_{lr}\;(\Xi$|$U>t_{2})$.*

**Proof.**  To prove (i), we must show that g(ξ| U>t)/g(ξ|U=t) is non-decreasing in ξ>0, for all t≥0. By (8) and (9),

$$\begin{array}{cc} \frac{g(\xi \;|\;U>t)}{g(\xi \;|\;U=t)}\; & =\frac{\bar{F}\left(t\;|\;\xi \right)\int_{0}^{\infty }f\left(t\;|\;\xi \right)g\left(\xi \right)\;d\xi }{f\left(t\;|\;\xi \right)\int_{0}^{\infty }\bar{F}\left(t\;|\;\xi \right)g\left(\xi \right)\;d\xi }\\ \; & =\frac{\left(v_{0}\left(t\right)-t/\xi \right)\int_{0}^{\infty }\left(\xi g\left(\xi \right)/\left(\xi v_{0}\left(t\right)-t\right)\right)\exp\left(-\int_{0}^{t}\frac{\xi v_{0}^{\prime }\left(x\right)}{\xi v_{0}\left(x\right)-x}dx\right)\;d\xi }{\int_{0}^{\infty }g\left(\xi \right)\exp\left(-\int_{0}^{t}\frac{\xi v_{0}^{\prime }\left(x\right)}{\xi v_{0}\left(x\right)-x}dx\right)\;d\xi }, \end{array}$$

which is non-decreasing in ξ>0, for all t≥0. In the case (ii), we must show that g(ξ|U>t) is TP_2_ in (ξ,t)∈(0,∞)×[0,∞). The desired conclusion holds if, and only if, $\left(\partial^{2}\right)/\left(\partial \xi \partial t\right)\ln(g(\xi$|U>t))≥0, for all ξ>0 and for all t≥0. By (9), we derive

$$\begin{array}{cc} \frac{\partial^{2}}{\partial t\partial \xi }\ln\left(\frac{\bar{F}\left(t\;|\;\xi \right)g\left(\xi \right)}{\int_{0}^{\infty }\bar{F}\left(t\;|\;\xi \right)g\left(\xi \right)\;d\xi }\right) & =\frac{\partial }{\partial \xi }\frac{\partial }{\partial t}\left(\ln\left(\frac{g(\xi )}{C(t)}\right)-\int_{0}^{t}\frac{\xi v_{0}^{\prime }\left(x\right)}{\xi v_{0}\left(x\right)-x}dx\right)\\ \; & =\frac{\partial }{\partial \xi }\left(\frac{\xi v_{0}^{\prime }\left(t\right)}{t-\xi v_{0}\left(t\right)}-\ln\left(C\left(t\right)\right)\right)\\ \; & =\frac{tv_{0}^{\prime }\left(x\right)}{{\left(t-\xi v_{0}\left(t\right)\right)}^{2}}\geq 0,\;\;\mathrm{for}\;\mathrm{all}\;t\geq 0,\;\xi >0, \end{array}$$

where $C\left(t\right)=\int_{0}^{\infty }g\left(\xi \right)\exp\left(-\int_{0}^{t}\frac{\xi v_{0}^{\prime }\left(x\right)}{\xi v_{0}\left(x\right)-x}dx\right)\;d\xi$ is the normalizing constant of density (9), which does not depend on ξ. The proof is complete. □

By Theorem 1, since the strongest stochastic order is the likelihood ratio order thus the hazard rate and the usual stochastic order is deduced. That is, it is a further conclusion of Theorem 1(i) that for all t≥0, $\left(\Xi |U=t\right)\leq_{hr}\;\left(\Xi |U>t\right)$, $\left(\Xi |U=t\right)\leq_{st}\;\left(\Xi |U>t\right)$, and a conclusion of Theorem 1(ii) that for all non-negative $t_{1}\leq t_{2}$, $\left(\Xi |U>t_{1}\right)\leq_{hr}\;\left(\Xi |U>t_{2}\right)$ and $\left(\Xi |U>t_{1}\right)\leq_{st}\;\left(\Xi |U>t_{2}\right)$. From relation (6) in Shrahili et al. [19], E(U|Ξ=ξ)=ξE(X), for all ξ>0. Thus, by the iterated expectation rule, E(U)=E(Ξ)E(X). However, one may be inquisitive about the relationship between the vitality function of *U* and the vitality function of *X*. The next result reveals their connection.

**Theorem** **2.** *Let U have survival function (6). Then, $v_{U}\left(t\right)=\frac{E[\Xi \bar{F}\left(t\;|\;\Xi \right)]}{E[\bar{F}\left(t\;|\;\Xi \right)]}v_{0}\left(t\right),$ for all t>0.*

**Proof.**  The vitality function of *U* is

$$\begin{array}{cc} v_{U}\left(t\right) & =\frac{\int_{t}^{\infty }\;uf_{U}\left(u\right)du}{{\bar{F}}_{U}\left(t\right)}\\ \; & =\frac{\int_{t}^{\infty }\;uE\left[f\left(u\;|\;\Xi \right)\right]du}{E[\bar{F}\left(t\;|\;\Xi \right)]}\\ \; & =\frac{E\left[\int_{t}^{\infty }\;uf\left(u\;|\;\Xi \right)du\right]}{E[\bar{F}\left(t\;|\;\Xi \right)]}\\ \; & =\frac{E\left[v\left(t\;|\;\Xi \right)\bar{F}\left(t\;|\;\Xi \right)\right]}{E[\bar{F}\left(t\;|\;\Xi \right)]}\\ \; & =\frac{E[\Xi \bar{F}\left(t\;|\;\Xi \right)]}{E[\bar{F}\left(t\;|\;\Xi \right)]}v_{0}\left(t\right),\;\;\mathrm{for}\;\mathrm{all}\;t\geq 0, \end{array}$$

ending the proof. □

The result of Theorem 2 discloses the closure property of the PVIT frailty model with respect to the mrl order. First, note that h(t|$\xi )=\xi v_{0}^{\prime }\left(t\right)/\left(\xi v_{0}\left(t\right)-t\right)$ is non-increasing in ξ>0, for all t≥0. This signifies that

(10) $$\left(U|\Xi =\xi_{1}\right)\leq_{hr}\left(U|\Xi =\xi_{2}\right),\mathrm{for}\;\mathrm{all}\xi_{1}\leq \xi_{2},$$

which further implies that (U|$\Xi =\xi_{1})\leq_{st}(U$|$\Xi =\xi_{2}),$ for all $\xi_{1}\leq \xi_{2}.$ Hence,

(11) $$\bar{F}\left(t|\xi \right)\mathrm{is}\;\mathrm{non-decreasing}\;\mathrm{in}\;\xi >0,\;\mathrm{for}\;\mathrm{all}\;t\geq 0.$$

Thus, by positive association principle, $Cov(\Xi ,\bar{F}(t$|Ξ))≥0, for all t≥0. From Theorem 2

$$\begin{array}{cc} \frac{v_{U}\left(t\right)}{v_{0}\left(t\right)} & =\frac{E[\Xi \bar{F}\left(t\;|\;\Xi \right)]}{E[\bar{F}\left(t\;|\;\Xi \right)]}\\ \; & \geq E(\Xi ),\;\;\mathrm{for}\;\mathrm{all}\;t\geq 0. \end{array}$$

By assumption, $v_{U}\left(t\right)\geq v_{0}\left(t\right),$ for all t≥0, or equivalently, $m_{U}\left(t\right)\geq m_{0}\left(t\right),$ for all t≥0.

In the framework of the PVIT frailty model, conditions under which *F* is less than $F_{U}$ in terms of some stochastic orders can be found.

**Theorem** **3.** *Let U follow the pdf (7) with the sf (6).*

*(i)*  *If P(Ξ≥1)=1, then $X\leq_{lr}U$ if, and only if, $v_{0}\left(t\right)/\sqrt{t}$ is non-decreasing.*
*(ii)*  *If E(Ξ)≥1, then $X\leq_{hr}U$ and thus $X\leq_{st}U$ and $X\leq_{mrl}U.$*

**Proof.**  (i) The hazard rate of *U* given Ξ=ξ is $h\left(t|\xi \right)=\xi v_{0}^{\prime }\left(t\right)/\left(\xi v_{0}\left(t\right)-t\right).$ From, (7) since P(Ξ≥1)=1 implies g(ξ)=0 for ξ<1 thus *U* has pdf

$$f_{U}\left(t\right)=\int_{1}^{\infty }h\left(t|\xi \right)e^{-\int_{0}^{t}h\left(x|\xi \right)dx}g\left(\xi \right)\;d\xi .$$

Note that *X* has pdf

$$f\left(t\right)=\int_{1}^{\infty }h\left(t|1\right)e^{-\int_{0}^{t}h\left(x|1\right)dx}g\left(\xi \right)\;d\xi .$$

Thus, we can write $f_{U}\left(t\right)/f\left(t\right)=E\left[\phi \left(t,\Xi \right)\right],$ where

$$\phi \left(t,\xi \right)=\frac{h(t|\xi )}{h(t|1)}e^{\int_{0}^{t}\left(h\left(x|1\right)-h\left(x|\xi \right)\right)dx}.$$

It is seen that ϕ(t,ξ) is non-decreasing in t>0 if, and only if,

$$\frac{h^{\prime }\left(t|\xi \right)}{h(t|\xi )}-h\left(t|\xi \right)\geq \frac{h^{\prime }\left(t|1\right)}{h(t|1)}-h\left(t|1\right),\;\mathrm{for}\;\mathrm{all}\;t>0\;\mathrm{and}\;\mathrm{for}\;\mathrm{all}\;\xi >1,$$

In this setting, one has

$$\begin{array}{cc} \frac{h^{\prime }\left(t|\xi \right)}{h(t|\xi )}-h\left(t|\xi \right) & =\frac{v_{0}^{\prime \prime }\left(t\right)}{v_{0}^{\prime }\left(t\right)}-\frac{v_{0}^{\prime }\left(t\right)-\frac{1}{\xi }}{v_{0}\left(t\right)-\frac{t}{\xi }}-\frac{v_{0}^{\prime }\left(t\right)}{v_{0}\left(t\right)-\frac{t}{\xi }}\\ \; & =\frac{v_{0}^{\prime \prime }\left(t\right)}{v_{0}^{\prime }\left(t\right)}-\frac{2v_{0}^{\prime }\left(t\right)-\frac{1}{\xi }}{v_{0}\left(t\right)-\frac{t}{\xi }}\\ \; & =\frac{v_{0}^{\prime \prime }\left(t\right)}{v_{0}^{\prime }\left(t\right)}+\frac{1-2\xi v_{0}^{\prime }\left(t\right)}{\xi v_{0}\left(t\right)-t}. \end{array}$$

It can be verified that

$$\left\{\frac{h^{\prime }\left(t|\xi \right)}{h(t|\xi )}-h\left(t|\xi \right)\right\}-\left\{\frac{h^{\prime }\left(t|1\right)}{h(t|1)}-h\left(t|1\right)\right\}=\frac{\left(\xi -1\right)(t\left(d/dt\right)\left(v_{0}\left(t\right)/\sqrt{t}\right))}{\left(\xi v_{0}\left(t\right)-t\right)\left(v_{0}\left(t\right)-t\right)}$$

Therefore, if $m_{0}$ is the mrl of *X* then

(12) $$\frac{d}{dt}\frac{f_{U}\left(t\right)}{f(t)}=t\frac{d}{dt}\frac{v_{0}\left(t\right)}{\sqrt{t}}\int_{1}^{\infty }\frac{\left(\xi -1)\phi (t,\xi \right)}{v\left(t|\xi \right)m_{0}\left(t\right)}g\left(\xi \right)d\xi$$

is non-negative if, and only if, $v_{0}\left(t\right)/\sqrt{t}$ is non-decreasing in t>0 as far as ξ≥1. (ii). We get

$$\begin{array}{cc} h_{U}\left(t\right) & =\frac{\int_{0}^{\infty }h\left(t|\xi \right)e^{-\int_{0}^{t}h\left(x|\xi \right)dx}g\left(\xi \right)d\xi }{\int_{0}^{\infty }e^{-\int_{0}^{t}h\left(x|\xi \right)dx}g\left(\xi \right)d\xi }\\ \; & =\int_{0}^{\infty }h\left(t|\xi \right)g^{*}\left(\xi |t\right)d\xi \\ \; & =E[h\left(t|\Xi^{*}\right)], \end{array}$$

where $\Xi^{*}$ is a non-negative random variable with pdf

$$g^{*}\left(\xi |\xi \right)=\frac{e^{-\int_{0}^{t}h\left(x|\xi \right)dx}g\left(\xi \right)}{\int_{0}^{\infty }e^{-\int_{0}^{t}h\left(x|\xi \right)dx}g\left(\xi \right)d\xi }=\frac{\bar{F}\left(t|\xi \right)g\left(\xi \right)}{E[\bar{F}\left(t|\Xi \right)]}.$$

The function ξ↦h(t|ξ) is twice differentiable with respect to ξ>0 and for all ξ>0,

$$\frac{\partial^{2}}{\partial \xi^{2}}h\left(t|\xi \right)=-\frac{2tv_{0}\left(t\right)v_{0}^{\prime }\left(t\right)}{{\left(\xi v_{0}\left(t\right)-t\right)}^{2}}\geq 0,\;\mathrm{for}\;\mathrm{all}\;t\geq 0.$$

Thus, −h(t|ξ) is convex in ξ and hence Jensen’s inequality yields

(13) $$\begin{array}{c} h_{U}\left(t\right)=E\left[h\left(t|\Xi^{*}\right)\right]\leq h\left(t|E\left(\Xi^{*}\right)\right),\;\mathrm{for}\;\mathrm{all}\;t\geq 0. \end{array}$$

Now, since from (11) $\bar{F}\left(t|\xi \right)$ is, for all t≥0, non-decreasing in ξ thus Ξ and $\bar{F}\left(t|\Xi \right)$ are positively associated and hence

$$E\left(\Xi^{*}\right)=\frac{E[\Xi \bar{F}\left(t|\Xi \right)]}{E[\bar{F}\left(t|\Xi \right)]}\geq E\left(\Xi \right)\geq 1.$$

Because h(t|ξ) is non-increasing in ξ for all t≥0 and since $h\left(t|1\right)=h_{0}\left(t\right)$, from (13) we conclude that

$$h_{U}\left(t\right)\leq h\left(t|E\left(\Xi^{*}\right)\right)\leq h\left(t|E\left(\Xi \right)\right)\leq h_{0}\left(t\right),forallt\geq 0.$$

□

**Remark** **2.** *In the proof of Theorem 3(i), because of (12), the shape each of the functions $v_{0}\left(t\right)/\sqrt{t}$ and $f_{U}\left(t\right)/f\left(t\right)$ for t≥0 exhibits is analogous. Therefore, a sufficient condition on the baseline vitality function, under which the graph of $f_{U}\left(t\right)/f\left(t\right)$ has a special behavior when $X\leq_{lr}U$ does not hold, can also be recognized as well as situations where the graph of it indicates an increasing behavior when $X\leq_{lr}U$.*

**Example** **1.** *Suppose that Ξ is a random variable with pdf $g\left(\xi \right)=1/\xi^{2},\xi \geq 1$. Let X have exponential distribution with mean 1/λ having vitality function $v_{0}\left(t\right)=t+1/\lambda$. Then, the random variable U given Ξ=ξ≥1 with sf*

(14) $$\bar{F}\left(t|\xi \right)={\left(\frac{1}{\frac{(\xi -1)\lambda t}{\xi }+1}\right)}^{\frac{\xi }{\xi -1}}$$

*has the vitality function $v\left(t|\xi \right)=\xi v_{0}\left(t\right)=\xi t+\xi /\lambda$. The unconditional random variable U has the pdf $f_{U}\left(t\right)=\int_{1}^{\infty }f\left(t|\xi \right)g\left(\xi \right)d\xi$. It can be seen that $v_{0}\left(t\right)/\sqrt{t}$ is non-increasing in t≤(1/λ) and is non-decreasing in t>(1/λ) and also that the function*

$$\frac{f_{U}\left(t\right)}{f(t)}=e^{\lambda t}\int_{1}^{\infty }{\left(\frac{1}{\frac{(\xi -1)\lambda t}{\xi }+1}\right)}^{1+\frac{\xi }{\xi -1}}\xi^{-2}d\xi$$

**Figure 1:**
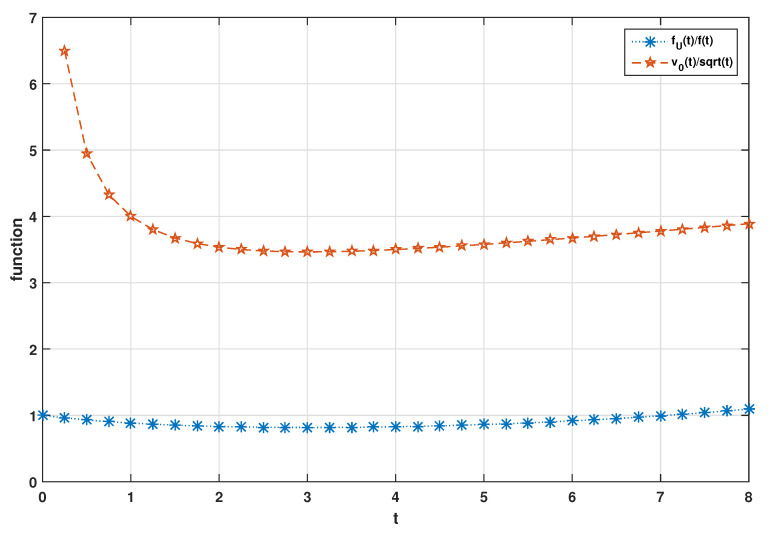
Plot of $f_{U}\left(t\right)/f\left(t\right)$ and $v_{0}\left(t\right)/\sqrt{t}$ when λ=1/3.

*follows a same behavior as shown in Figure 1 for some given λ as mentioned in Remark 2. Further,*

$$h_{U}\left(t\right)=\frac{\int_{1}^{\infty }{\left(\frac{1}{\frac{(\xi -1)\lambda t}{\xi }+1}\right)}^{1+\frac{\xi }{\xi -1}}\lambda \xi^{-2}d\xi }{\int_{1}^{\infty }{\left(\frac{1}{\frac{(\xi -1)\lambda t}{\xi }+1}\right)}^{\frac{\xi }{\xi -1}}\xi^{-2}d\xi }.$$

**Figure 2:**
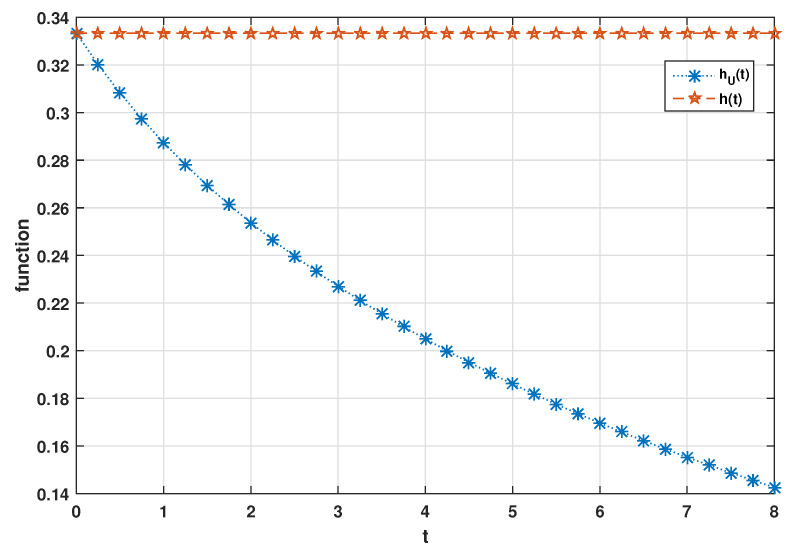
Plot of $h_{U}\left(t\right)$ and h(t) when λ=1/3.

*As shown in Figure 2 for some λ>0 that $h_{U}\left(t\right)\leq h\left(t\right)$ for t≥0 in which h(t)=λ for all t≥0. This validates the result of Theorem 3(ii).*

It is quite useful to predict the survival function of *U* given Ξ=ξ through some bounds for it. For the early interval time (0,ξE(X)) of life of individuals with the certain vitality growth ξ, we can get the following lower bound for $\bar{F}\left(t|\xi \right).$

**Theorem** **4.** *Let T with sf $\bar{F}\left(\cdot |\xi \right)$ as given in (4) and X with sf $\bar{F}$ have proportional vitality functions. Then,*

$$\bar{F}\left(t|\xi \right)\geq \frac{\xi E(X)-t}{\xi v_{0}\left(t\right)-t},\;\;for\;t<\xi E\left(X\right).$$

**Proof.**  The function $\phi \left(u\right)={\left(u-t\right)}_{+}=\max\left\{u-t,0\right\}$ is a convex function in *u* for all t. Thus, on one hand, by Jensen’s inequality

$$\begin{array}{cc} E{\left(T-t\right)}_{+} & \geq {\left(E\left(T\right)-t\right)}_{+}\\ \; & ={\left(\xi E\left(X\right)-t\right)}_{+},\;t\geq 0. \end{array}$$

On the other hand, since T−t and I[T>t] are two positively associated random variables,

$$\begin{array}{cc} E{\left(T-t\right)}_{+} & =E((T-t)I[T>t])\\ \; & \geq \left(E\left(T\right)-t\right)\bar{F}\left(t|\xi \right)\\ \; & =\left(\xi E\left(X\right)-t\right)\bar{F}\left(t|\xi \right),\;t\geq 0. \end{array}$$

Therefore, for all t≥0, we have

$$\begin{array}{cc} E{\left(T-t\right)}_{+} & =\left(\xi v_{0}\left(t\right)-t\right)\bar{F}\left(t|\xi \right)\\ \; & \geq \left(\xi E\left(X\right)-t\right)\max\{\bar{F}\left(t|\xi \right),I\left[\xi E\left(X\right)>t\right]\}, \end{array}$$

which concludes the result for all t<ξE(X). □

The following result presents an upper bound for $\bar{F}\left(t|\xi \right).$

**Theorem** **5.** *If T and X have proportional vitality functions, then*

$$\bar{F}\left(t|\xi \right)\leq \frac{Var(T)}{Var\left(T\right)+\xi^{2}{\left(v_{0}\left(t\right)-E\left(X\right)\right)}^{2}},$$

*in which $Var\left(T\right)=E\left(U^{2}|\Xi =\xi \right)-\xi^{2}E^{2}\left(X\right)$.*

**Proof.**  By Cauchy–Schwarz’s inequality,

$$\begin{array}{cc} Cov(T-t,I[T>t]) & =E\left[\left(T-t\right)I\left[T>t\right]\right]-\left(E\left(T\right)-t\right)\bar{F}\left(t|\xi \right)\\ \; & \leq \sqrt{Var(T)}\sqrt{Var(I[T>t])} \end{array}$$

Thus,

$$\begin{array}{cc} \left(\xi v_{0}\left(t\right)-t\right)\bar{F}\left(t|\xi \right) & =E{\left(T-t\right)}_{+}\\ \; & =E[(T-t)I[T>t]]\\ \; & \leq \left(E\left(T\right)-t\right)\bar{F}\left(t|\xi \right)+\sqrt{Var(T)}\sqrt{\bar{F}\left(t|\xi \right)\left(1-\bar{F}\left(t|\xi \right)\right)}. \end{array}$$

The result follows by doing some simple calculation. □

**Figure 3:**
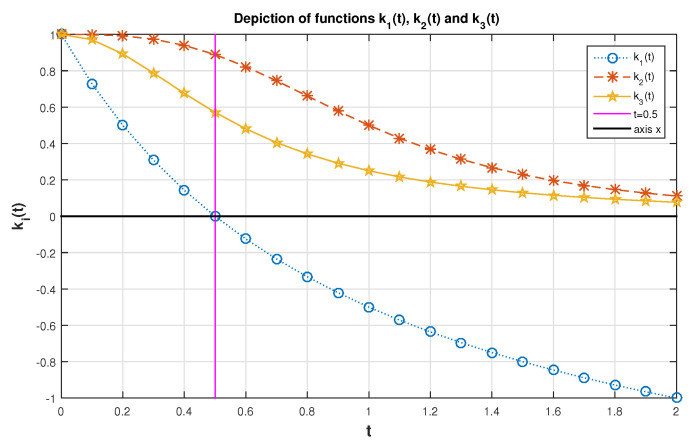
Plot of the functions $k_{i}\left(t\right),i=1,2,3$.

**Example** **2.** *Let T have the sf (14) with ξ=3/2. By Theorem 4, $\bar{F}\left(t|\xi \right)\geq \left(1-2t\right)/\left(1+t\right)$ for t<0.5. From Theorem 5 $\bar{F}\left(t|\xi \right)\leq 1/\left(1+3t^{2}\right)$ for all t≥0. Figure 3 clarifies this issue.*

$$k_{1}\left(t\right)=\frac{1-2t}{1+t},\;\;\;k_{2}\left(t\right)=\frac{1}{1+3t^{2}},\;\;\;k_{3}\left(t\right)=\bar{F}\left(t|\frac{3}{2}\right)=\frac{1}{{\left(1+t\right)}^{3}}.$$

## 4. PVIT Model with Time-Dependent Constant of Proportionality

It was argued by Shrahili et al. [19] that the relationship in (3) does not hold if the functions v(·|ξ) and $v_{0}$ are associated with two lifetime distributions with either (eventually) IFR property or (eventually) DMRL property. This, in turn, is a limitation for the usability of the model since the PVIT model cannot be applied to model distributions that induce a positive aging path. The deficiency of fitting the model on (ultimately) IFR and (ultimately) DMRL distributions is mainly due to leaving out the possibility of having a time-dependent proportionality parameter in the model. To eliminate this blind spot and to entertain more practical situations, the model (3) can be developed into the case where ξ possibly varies over an interval of time and also ξ hinges at some parameters.

Suppose that random lifetimes *X* and *Y* have vitality functions V(·|θ) and $V_{0}\left(\cdot \right)$, where $\theta ={\left(\theta_{1},\ldots ,\theta_{m}\right)}^{T}$ is a vector of parameters in which $\theta_{i}$’s are all real-valued. Then, the random variables *X* and *Y* are said to have time-dependent proportional vitalities model when the relationship between V(·|θ) and $V_{0}\left(\cdot \right)$ is expressed by

(15) $$V\left(t|\theta \right)=\xi \left(t,\theta \right)V_{0}\left(t\right),\mathrm{for}\mathrm{all}t\geq 0,$$

ξ:(t,θ)↦ξ(t,θ) is a non-stochastic and non-negative function. It is further assumed that ξ is a smooth function of *t* and $\theta_{k},\;k=1,2,\ldots ,m$ so that the partial derivatives exist and are also continuous. From the relationship (1), the expression in (15) can be rewritten based on mrl functions of *X* and *Y* as

$$M\left(t|\theta \right)=\xi \left(t,\theta \right)M_{0}\left(t\right)+\left(\xi \left(t,\theta \right)-1\right)t,\mathrm{for}\mathrm{all}t\geq 0,$$

through which the survival function of *Y* is obtained as

(16) $$\bar{F}\left(t|\theta \right)=\exp\left(-\int_{0}^{t}\frac{d\{\xi \left(x,\theta \right)V_{0}\left(x\right)\}/dx}{\xi \left(x,\theta \right)V_{0}\left(x\right)-x}dx\right).$$

The function $\bar{F}\left(\cdot |\theta \right)$ in (16) is a valid survival function if the following conditions hold:

(i) For all x≥0, $\xi \left(x,\theta \right)\geq x/V_{0}\left(x\right).$
(ii) $\int_{0}^{t}\frac{d\{\xi \left(x,\theta \right)V_{0}\left(x\right)\}/dx}{\xi \left(x,\theta \right)V_{0}\left(x\right)-x}dx<\infty ,$ for all 0≤t<∞.
(iii) $\int_{0}^{\infty }\frac{d\{\xi \left(x,\theta \right)V_{0}\left(x\right)\}/dx}{\xi \left(x,\theta \right)V_{0}\left(x\right)-x}dx=\infty .$

The choice of the function ξ may be controversial in applied data analysis as well as in theoretical studies. From conditions (i)–(iii) inserted above, the choice of ξ in order to decide among various forms depends on the appearance of $V_{0}.$ However, the baseline vitality function $V_{0}$ may be considered to be known or unknown. We convert the model from its current form in (4) into a regression model. Let us assume, specifically, that

zi(t)=zi[1]zi[2](t),andγ=αβ

are, respectively, the vector of covariates composed into time-dependent and time-free sections for the *i*th individual so that

$$z_{i}^{\left[1\right]}={\left(\zeta_{1i}^{\left[1\right]},\ldots ,\zeta_{pi}^{\left[1\right]}\right)}^{T}\;\;\mathrm{and}\;\;z_{i}^{\left[2\right]}\left(t\right)={\left(z_{1i}^{\left[2\right]}\left(t\right),\ldots ,z_{li}^{\left[2\right]}\left(t\right)\right)}^{T}$$

and the vector of regression coefficients. In fact, $\alpha^{T}={\left(\alpha_{1},\ldots ,\alpha_{p}\right)}^{T}$ is the prognostic covariate for subject (or individual) *i*, including *p* factors describing that individual in the analysis independently of time, and $\beta^{T}={\left(\beta_{1},\ldots ,\beta_{l}\right)}^{T}$ describes regression coefficient of the time-dependent part of the covariates. It is clear that the failure of an individual at time *t* is not only affected by measuring a risk factor at time *t* but it is, rationally, a result of the amount of the related covariate evaluated up to the time t. For this reason, it is natural to integrate the amounts of an instantaneous covariate over the interval (0,t], to have an overall value for the associated covariate effect, such that

$$z_{i}^{\left[2\right]}\left(t\right)={\left(\int_{0}^{t}\zeta_{1i}^{\left[2\right]}\left(s\right)\;ds,\ldots ,\int_{0}^{t}\zeta_{li}^{\left[2\right]}\left(s\right)\;ds\right)}^{T}$$

where $\zeta_{ki}^{\left[2\right]}\left(s\right)$ is the instantaneous status of the *i*th individual in the factor *k* of the covariate at time s∈(0,t] and $\zeta_{ki}^{\left[1\right]}$ is that part of the effect of the *k*th covariate in the *i*th individual, which enhances/diminishes the vitality function as independent of time. In some way, the time-free effect of the covariate can represent a measure of the amount of susceptibility in individuals to cause of the event. Note that if $z_{i}^{\left[2\right]}\left(t\right)={\left(z_{1i}^{\left[2\right]}\left(t\right),\ldots ,z_{li}^{\left[2\right]}\left(t\right)\right)}^{T}$ then $\zeta_{ki}^{\left[2\right]}\left(t\right)=dz_{ki}^{\left[2\right]}\left(t\right)/dt$, for all k=1,2,…,l and i=1,2,…,n. Therefore, a natural way for robust and complete assessment of covariate effects is provided. The idea for dividing the covariates into two groups, namely time-dependent and time-free ones, could be convinced so that in practical situations, some events are influenced by covariates progressively over time, e.g., the effect of sport on the treatment of a disease, while the others may be due to the origin of some covariates, for example, sex or blood type of an individual, which does not change over time. To explain more, the risk of failure of an individual, caused by *k*th covariate factor, at time *t* is not only due to some risk at time t, but all in interval time (0,t]. For example, suppose for an individual *i* who died of lung cancer at time $t_{i}$, $z_{i}\left(t_{i}\right)$ is the covariate factor that relates the death of the person to smoking. It is obvious that he/she has not been a cigarette smoker since the time of their birth and also if he/has started to consume cigarettes at time τ(<t), then its intensity could be variable over the interval (τ,t), which is why $\zeta_{ki}^{\left[2\right]}\left(t\right)$ works in practice. In parallel, $\zeta_{ki}^{\left[1\right]}$ can show the part of the effect of the covariate $z_{i}\left(t\right)$ that remains unexplained by observed covariate $\zeta_{ki}^{\left[2\right]}\left(t\right)$. Then, an appropriate flexible choice of ξ would be as follows:

$$\begin{array}{cc} \xi (t,\gamma ) & =\exp(\gamma^{T}z_{i}\left(t\right))\\ \; & =\exp(\sum_{k=1}^{p}\alpha_{k}\zeta_{ki}^{\left[1\right]}+\sum_{k=1}^{l}\beta_{k}\int_{0}^{t}\zeta_{ki}^{\left[2\right]}\left(s\right)ds). \end{array}$$

Suppose that $Y_{i}$ for i=1,2,…,n is the event time of the *i*th subject, which is realized to be equal with $t_{i}$ after observing the sample. The time-dependent PVIT model then requires

(17) $$V_{i}\left(t_{i}|\gamma \right)=\exp\left(\gamma^{T}z_{i}\left(t\right)\right)V_{0}\left(t_{i}\right),$$

where $\gamma^{T}=\left(\gamma_{1}\left(t\right),\ldots ,\gamma_{m}\left(t\right)\right)$, where m=p+l. To derive the associated likelihood function for the observed sample, we shall take $Y_{1}$, $Y_{2},\ldots$,$Y_{n}$ as independent times-to-event, which are not necessarily identical in distribution.

## 5. Inference about γ When $V_{0}$ Is Known

To estimate the parameters of the model (2.3), α and β, we proceed here with the method of maximum likelihood. Suppose that for all individuals in the sample with amounts of $t_{1},t_{2},\ldots ,t_{n}$ for the response variable, the values of $(z_{i}^{\left[1\right]},z_{i}^{\left[2\right]}\left(x\right)),$ for all $x\in (0,t_{i}],\;i=1,2,\ldots ,n$, are available or can be computed. In the model (2.3), if $V_{0}\left(t\right)$ is specified completely or up to some unknown parameters, and if, furthermore, the function given in (17) is valid as the vitality function of a random variable, then the model (2.3) is fully parametric. Suppose that *X* has an absolutely continuous distribution with vitality function $V_{0}.$ The likelihood function corresponding to the model (17) is given by

$$\begin{array}{ccc} L(\alpha ,\beta ) & = & \Pi_{i=1}^{n}f_{i}\left(t_{i}|\alpha ,\beta ,z_{i}^{\left[1\right]},z_{i}^{\left[2\right]}\left(t_{i}\right)\right)\\ & = & \Pi_{i=1}^{n}\frac{V_{0}^{{}^{\prime }}\left(t_{i}\right)+\beta^{T}\zeta_{i}^{\left[2\right]}\left(t_{i}\right)V_{0}\left(t_{i}\right)}{V_{0}\left(t_{i}\right)-t_{i}e^{-\alpha^{T}z_{i}^{\left[1\right]}-\beta^{T}z_{i}^{\left[2\right]}\left(t_{i}\right)}}\;e^{-\int_{0}^{t_{i}}\frac{V_{0}^{{}^{\prime }}\left(x\right)+\beta^{T}\zeta_{i}^{\left[2\right]}\left(x\right)V_{0}\left(x\right)}{V_{0}\left(x\right)-xe^{-\alpha^{T}z_{i}^{\left[1\right]}-\beta^{T}z_{i}^{\left[2\right]}\left(x\right)}}dx}, \end{array}$$

where $\zeta_{i}^{\left[2\right]}\left(t\right)={\left(\zeta_{1i}^{\left[2\right]}\left(t\right),\ldots ,\zeta_{li}^{\left[2\right]}\left(t\right)\right)}^{T}$ and $V_{0}^{{}^{\prime }}$ is the derivative of $V_{0}.$ To derive likelihood equations, we first get the log-likelihood function. Taking differentiation of ln(L(α,β)) with respect to $\alpha_{k},$ for k=1,2,…,p and also with respect to $\beta_{k},$ for k=1,2,…,l provides, respectively, the equations

$$\sum_{i=1}^{n}\int_{0}^{t_{i}}\frac{x\left(V_{0}^{{}^{\prime }}\left(x\right)+\beta^{T}\zeta_{i}^{\left[2\right]}\left(x\right)V_{0}\left(x\right)\right)\zeta_{ki}^{\left[1\right]}e^{-\alpha^{T}z_{i}^{\left[1\right]}-\beta^{T}z_{i}^{\left[2\right]}\left(x\right)}}{{\left(V_{0}\left(x\right)-xe^{-\alpha^{T}z_{i}^{\left[1\right]}-\beta^{T}z_{i}^{\left[2\right]}\left(x\right)}\right)}^{2}}dx$$

$$-\sum_{i=1}^{n}\frac{\zeta_{ki}^{\left[1\right]}e^{-\alpha^{T}z_{i}^{\left[1\right]}-\beta^{T}z_{i}^{\left[2\right]}\left(t_{i}\right)}}{V_{0}\left(t_{i}\right)-t_{i}e^{-\alpha^{T}z_{i}^{\left[1\right]}-\beta^{T}z_{i}^{\left[2\right]}\left(t_{i}\right)}}=0,\;k=1,2,\ldots ,p;$$

and the equations

$$\sum_{i=1}^{n}\frac{\zeta_{ki}^{\left[2\right]}\left(t_{i}\right)V_{0}\left(t_{i}\right)}{V_{0}^{{}^{\prime }}\left(t_{i}\right)+\beta^{T}\zeta_{i}^{\left[2\right]}\left(t_{i}\right)V_{0}\left(t_{i}\right)}-\sum_{i=1}^{n}\frac{t_{i}\zeta_{ki}^{\left[2\right]}\left(t_{i}\right)}{V_{0}\left(t_{i}\right)e^{\alpha^{T}z_{i}^{\left[1\right]}+\beta^{T}z_{i}^{\left[2\right]}\left(t_{i}\right)}}$$

$$-\sum_{i=1}^{n}\int_{0}^{t_{i}}\frac{\zeta_{ki}^{\left[2\right]}\left(x\right)V_{0}\left(x\right)\left(V_{0}\left(x\right)-xe^{-\alpha^{T}z_{i}^{\left[1\right]}-\beta^{T}z_{i}^{\left[2\right]}\left(x\right)}\right)}{{\left(V_{0}\left(x\right)-xe^{-\alpha^{T}z_{i}^{\left[1\right]}-\beta^{T}z_{i}^{\left[2\right]}\left(x\right)}\right)}^{2}}dx$$

$$+\sum_{i=1}^{n}\int_{0}^{t_{i}}\frac{x\zeta_{ki}^{\left[2\right]}\left(x\right)e^{-\alpha^{T}z_{i}^{\left[1\right]}-\beta^{T}z_{i}^{\left[2\right]}\left(x\right)}\left(V_{0}^{{}^{\prime }}\left(x\right)+\beta^{T}\zeta_{i}^{\left[2\right]}\left(x\right)V_{0}\left(x\right)\right)}{{\left(V_{0}\left(x\right)-xe^{-\alpha^{T}z_{i}^{\left[1\right]}-\beta^{T}z_{i}^{\left[2\right]}\left(x\right)}\right)}^{2}}dx=0,$$

for k=1,2,…,l. The above equations can be solved using the numerical iteration method of Newton–Raphson.

## 6. Concluding Remarks

In this paper, the proportional vitalities model in Shrahili et al. [19] is exposed to random effects of the vitality growth parameter for which several characteristics of the model and further stochastic ordering results have been obtained. To study the association between the amount of the vitality growth parameter and the resulting cdf in the dynamic version of the model, the random behavior of the vitality growth parameter and its influence on the resultant random variable are evaluated.

Referring to situations in which the vitality growth parameter changes and fluctuates with time, the proportional vitalities model with the time-dependent parameter was considered. To present a method for statistical inference of the parameters of this model, the likelihood function of the sample of observations, including lifetimes and dynamic covariates, and resulting likelihood equations were derived. The numerical iteration method of Newton–Raphson was suggested to solve the likelihood equations.

In the context of proportional models, Shanthikumar [3] and also Kundu et al. [29] used the property of majorization (which is a useful notion in the context of statistical quantum mechanics) of the vectors of constants of proportionality in the proportional equilibrium rates model for two independent vectors satisfying the model, and several inequalities of expectations of Schur-convex (concave) functions were obtained. In the future study of this work, a similar strategy to study the possibility of comparison of random vectors with the PVIT model using the theory of majorization can be employed. To study aging aspects in the dynamic proportional vitalities model, a further study can be carried out. The authors are also interested in developing statistical analysis of the model when real data sets are available to show the performance of the method of estimation presented in the current work.

## Data Availability

No new data were created or analyzed in this study. Data sharing is not applicable to this article.
